# Crystal structure and features of 3′,8-di­benzyl­idene-4a,5,6,7,8,8a-hexa­hydro-2′*H*-spiro­[chromene-2,1′-cyclo­hexa­n]-2′-one

**DOI:** 10.1107/S2056989017014165

**Published:** 2017-10-06

**Authors:** Alexander Anis’kov, Vyacheslav Grinev, Irina Klochkova

**Affiliations:** a Institute of Chemistry, National Research Saratov State University, 83 Astrakhanskaya St., Saratov 410012, Russian Federation

**Keywords:** X-ray structural analysis, crystal structure, non-covalent inter­actions, spiro heterocycle

## Abstract

The synthesis and crystal structure of the title compound, C_28_H_28_O_2_, are reported. The C=C—C—C torsion angles in the phenyl­methyl­idene units are 166.6 (3) and −48.0 (4)°. In the crystal, mol­ecules form a three-dimensional network by means of weak C—H⋯O hydrogen bonds. The most important contributions to the crystal structure are the H⋯H inter­actions (68.8%), while the H⋯O contacts account for 4.5%.

## Abstract 2

In the title compound, the C=C—C—C torsion angles in the phenyl­methyl­idene units are 166.6 (3) and −48.0 (4)°. In the crystal, mol­ecules form a three-dimensional network by means of weak C—H⋯O hydrogen bonds. The most important contributions to the crystal structure are the H⋯H inter­actions (68.8%).

## Chemical context   

Spiro heterocycles are of great inter­est for the creation of new promising biologically active compounds. The spiro center causes a rigid, spatially oriented configuration, which makes the compounds containing them potentially more complementary to binding sites for biological targets (Mirzabekova *et al.*, 2008 ▸; Abou-Elmagd & Hashem, 2016 ▸; Saraswat *et al.*, 2016 ▸). A convenient way obtain heterocyclic compounds, including those with the spiro chromane moiety, is dimerization of Mannich ketones (Shchekina *et al.*, 2017 ▸).
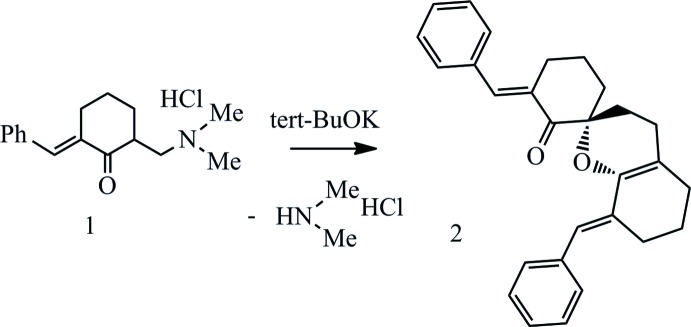


## Structural commentary   

**Figure 1:**
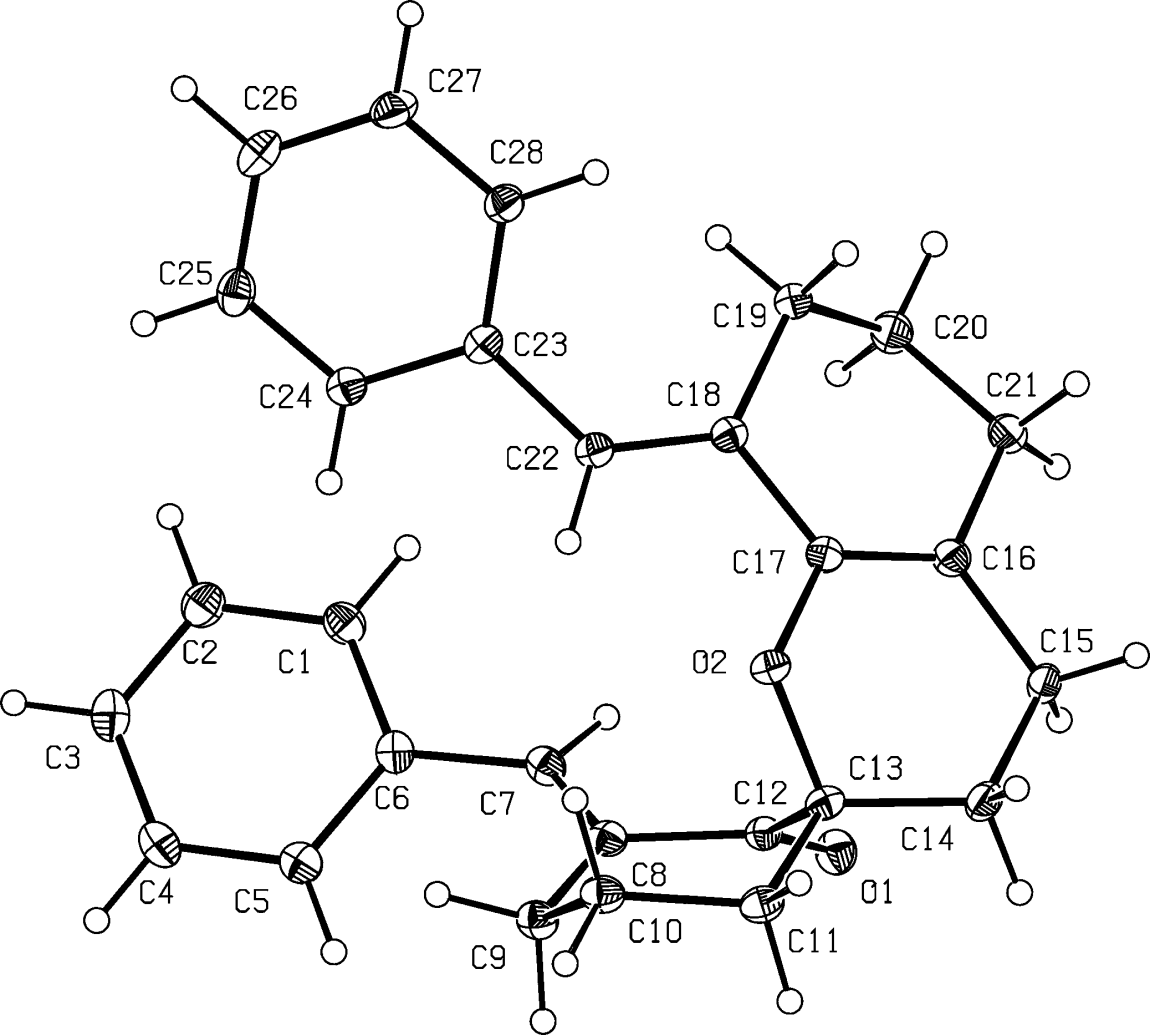
The mol­ecular structure of the title compound with atom-labeling scheme, with displacement ellipsoids drawn at the 50% probability level.

The structure of the title compound is shown in Fig. 1 ▸. The pyran, cyclo­hexa­none and methyl­ene­cyclo­hexene units are each non-planar structures with the following puckering parameters: *Q* = 0.447 Å, *θ* = 128.1°, *φ* = 249.3°; *Q* = 0.517 Å, *θ* = 167.2°, *φ* = 12.9°; and *Q* = 0.460 Å, *θ* = 130.0°, *φ* = 39.9°, respectively. In the two phenyl­methyl­idene moieties, the corresponding σ-bonds are shortened [C6—C7 = 1.475 (4) and C23—C22 = 1.471 (4) Å], which allows us to speak of incomplete π–π conjugation of aromatic rings and double bonds. These values are slightly longer than the bond lengths characteristic for complete conjugation in similarly constructed moieties (Golikov *et al.*, 2006 ▸); in particular, for di­benzyl­idene­cyclo­hexa­none it is 1.341 Å. The torsion angles C8=C7—C6—C5 and C18=C22—C23—C28 are similar [−38.5 (5) and −36.3 (5)°, respectively], and reflect the non-coplanarity of the phenyl­methyl­idene moiety, and therefore confirms incomplete conjugation of the phenyl and yl­idene moieties (Kriven’ko *et al.*, 2005 ▸). The values noted above significantly exceed the corresponding ones for torsion angles in analogous moieties in di­benzyl­idene cyclo­hexa­nones (−28.70°; Jia *et al.*, 1989 ▸). Such a significant deviation of the torsion angle from the expected value is probably due to van der Waals repulsion of hydrogen atoms on the cyclo­hexene atoms C9 and C19 and hydrogen atoms of the aromatic rings. Thus, the inter­atomic distance between the hydrogen atoms of the aromatic substituent at C5 and the methyl­ene group at C9 is 2.27 Å, close to the sum of the van der Waals radii for hydrogen atoms (2.2 Å). The C7=C8 bond is a little shorter than the C18=C22 bond [1.337 (4) and 1.346 (4) Å, respectively]. We believe that this is due to better conditions for π–π conjugation of the *Ph*–C22=C18—C17=C16 unit compared to the *Ph*—C7=C8—C12=O1 unit. So, the value of the C22=C18—C17=C16 torsion angle is 166.6 (3)° in comparison with 135.0 (3)° for C7=C8—C12=O1, allowing us to conclude a more pronounced flat structure for the former unit. The O2—C17 bond is noticeably shorter [1.391 (3) Å] than O2—C13 [1.446 (3) Å] due to conjugation of the endocyclic oxygen atom and a multiple bond. The bond lengths of the spiro center are within expected values, and are typical of those in similar moieties (Clark *et al.*, 2005 ▸; Kia *et al.*, 2012 ▸).

## Supra­molecular features   

**Figure 2:**
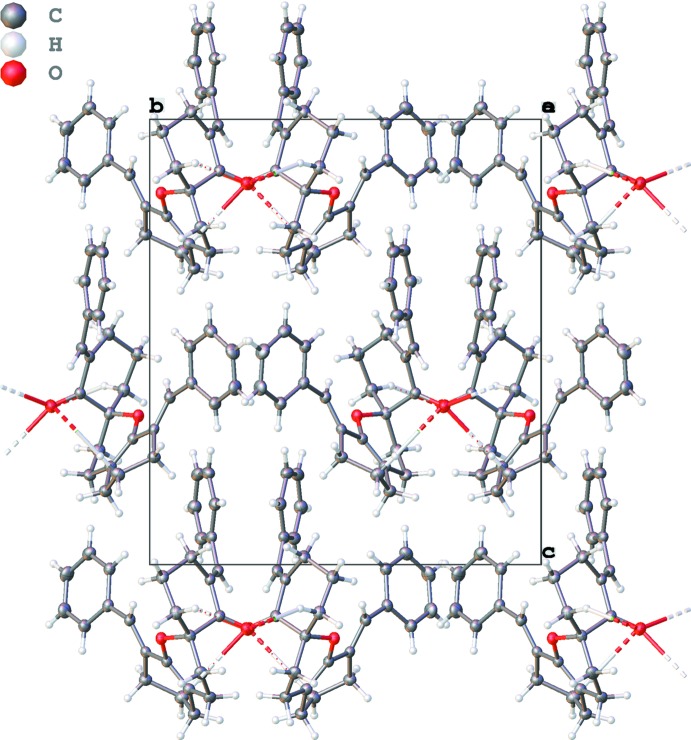
Graphical representation of the hydrogen bonds (dashed lines) along the *a* axis.

In the crystal, the mol­ecules are linked into a complex three-dimensional network by means of weak C20—H20*B*⋯O1^i^ and C11—H11*B*⋯O1^i^ hydrogen bonds between (Figs. 2 ▸–4 ▸ ▸ and Table 1 ▸).

## Analysis of the Hirshfeld Surfaces   

**Figure 5:**
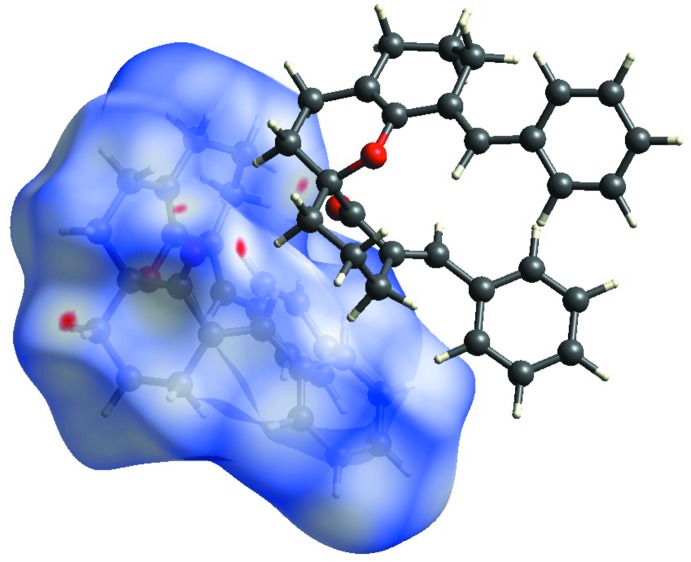
Graphical representation of the Hirshfeld surface mapped over *d*_norm_. The highlighted red spots on the top face of the surfaces indicate contact points with the atoms participating in the C—H⋯O inter­molecular inter­actions.

**Figure 6:**
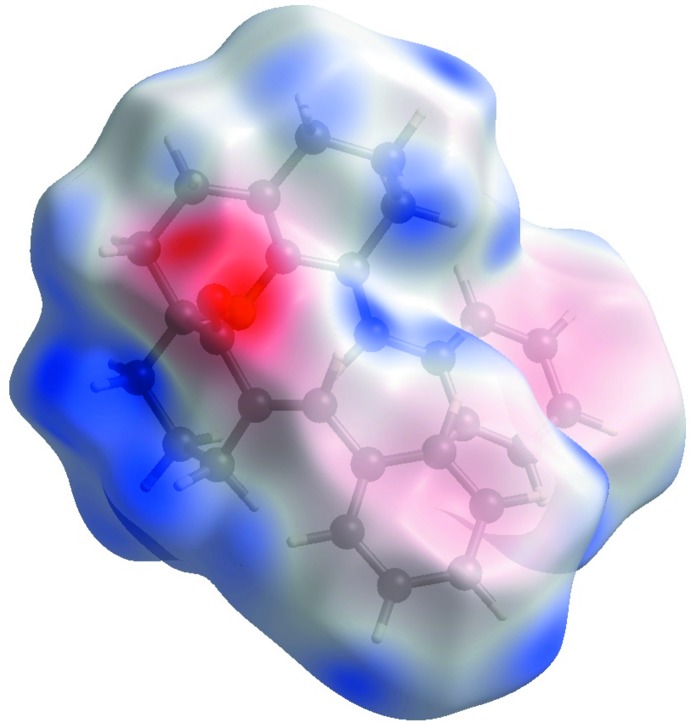
Graphical representation of the electrostatic potential surfaces.

**Figure 7:**
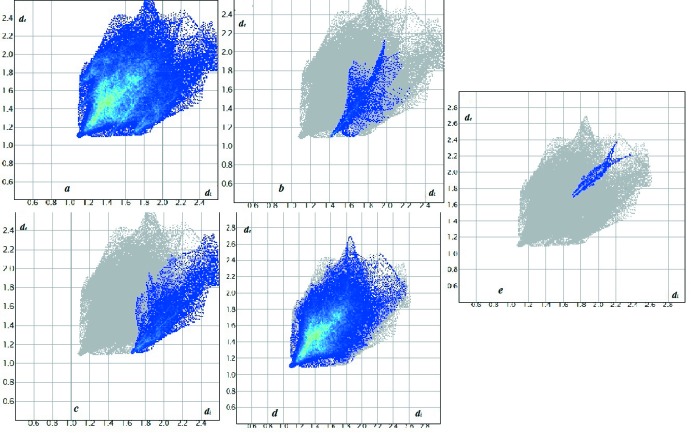
Graphical representation of the Hirshfeld surface two-dimensional fingerprint plot for the title compound (*a*) showing the: (*b*) H⋯O, (*c*) C⋯H, (*d*) H⋯H, (*e*) C⋯C inter­actions.

The C11—H11*B*⋯O1^i^ and C20—H20*B*⋯O1^i^ inter­actions are visualized as bright-red spots between the corresponding donor and acceptor atoms on the Hirshfeld surfaces, mapped by *d*_norm_ (Fig. 5 ▸). This is confirmed by the Hirshfeld surfaces, displayed as the electrostatic potential (Fig. 6 ▸), showing a negative potential around the oxygen atoms in the form of light-red clouds and a positive potential around the H atoms in the form of bluish clouds. The H⋯O contacts account for about 4.5% of the Hirshfeld surface displayed on the fingerprint plots with a curved surface with *d_e_* + *d_i_* ∼2.2 Å (Fig. 7 ▸). The largest proportion, 68.8%, is for H⋯H contacts, with a bright splash on the fingerprint plot corresponding to *d_e_* + *d_i_* ∼2.2 Å. The C⋯H inter­action corresponds to 12.2% *d_e_* + *d_i_* ∼2.4 Å with peaks in the region of the aromatic rings (Fig. 7 ▸). The presence of π–π stacking reflects the presence of C⋯C contacts, which account for only 1.0% of the Hirschfield surface with *d_e_* + *d_i_* ∼2.2 Å.

## Database survey   

The structure and configuration of the mol­ecule is complex and includes a spiro node and aryl­methyl­idene moieties. A similar spiro ring based on the Mannich ketone was described earlier (Siaka *et al.*, 2012 ▸). The tetra­hydro­pyridine ring is in an unsymmetrical half-chair conformation, while the cyclo­hexa­diene and cyclo­hexene rings display semi-boat conformations.

## Synthesis and crystallization   

A 5% solution of potassium *tert*-butoxide in *i*-iso­propanol (5 mL) was added to a 2-[(di­methyl­amino)­meth­yl)]-6-(phenyl­methyl­idene)cyclo­hexa­none solution (1.396 g, 5 mmol) in *i*-iso­propanol. The mixture was refluxed for two h, then cooled. The precipitated crystalline substance was washed with a 2% aqueous solution of acetic acid, recrystallized from *i*-iso­propanol, yielding colourless crystals (1.47 g, 74%), m.p. 413–414 K (*i*-*Pr*OH). ^1^H NMR (CDCl_3_): δ 1.56–1.83 (*m*, 4H, CH_2_), 1.90–2.30 (*m*, 1H, CH_2_), 2.61 (*tt*, 2H, *J* = 15.4, 7.8 Hz, CH_2_), 2.76–2.88 (*m*, 1H, CH_2_), 2.91–3.01 (*m*,1H, CH_2_), 6.81 (*s*, 1H, =CH), 7.10–7.41 (*m*, 11H, *Ar*, =CH). ^13^C NMR (CDCl_3_): δ 19.6, 22.9 23.8, 27.4, 27.8, 28.7, 29.6, 34.8, 78.9 (*spiro* C), 111.7, 120.3, 125.8, 127.8, 128.3, 129.3, 129.9, 130.1, 132.7, 134.7, 135.8, 138.0, 138.2, 143.2, 201.2 (C=O). Analysis calculated for C_28_H_28_O_2_ (396.2): C 73.23; H 5.23; N 6.32. Found: C 73.68; H 5.09; N 6.27.

## Refinement   

Crystal data, data collection and structure refinement details are summarized in Table 2 ▸.

## Supplementary Material

Crystal structure: contains datablock(s) I. DOI: 10.1107/S2056989017014165/rk2438sup1.cif

Structure factors: contains datablock(s) I. DOI: 10.1107/S2056989017014165/rk2438Isup2.hkl

CCDC reference: 1577738

Additional supporting information:  crystallographic information; 3D view; checkCIF report

## Figures and Tables

**Figure 3:**
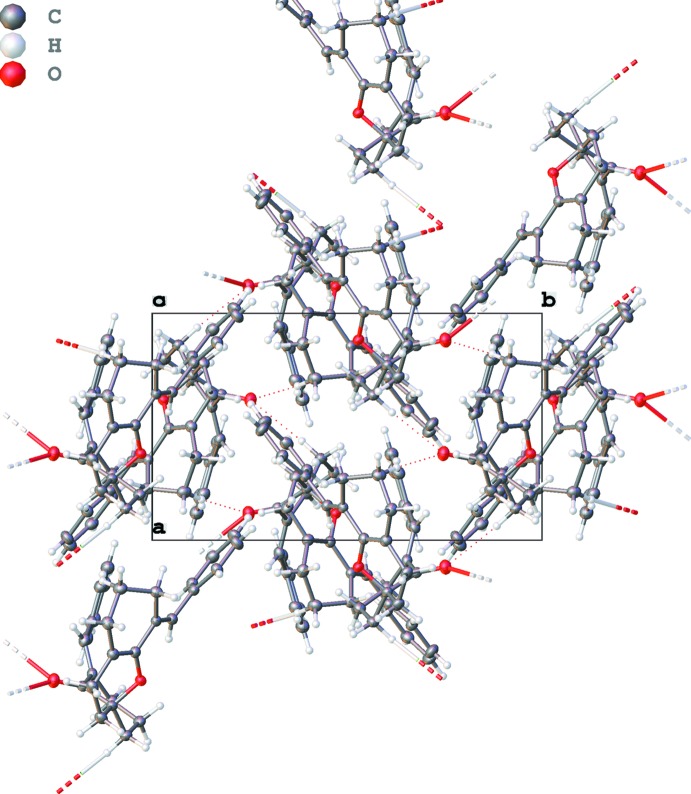
Graphical representation of the hydrogen bonds (dashed lines) along the *c* axis.

**Figure 4:**
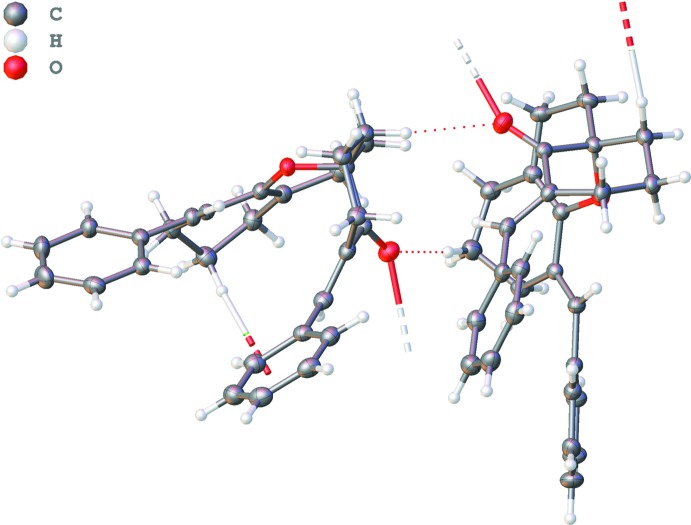
Graphical representation of the hydrogen bonds.

**Table 1:** Hydrogen-bond geometry (Å, °)

*D*—H⋯*A*	*D*—H	H⋯*A*	*D*⋯*A*	*D*—H⋯*A*
C20—H20*B*⋯O1^i^	0.99	2.64	3.630	175
C11—H11*B*⋯O1^i^	0.99	2.61	3.521	153

Symmetry code: (i) 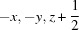.

**Table 2:** Experimental details

Crystal data	
Chemical formula	C_28_H_28_O_2_
*M*_r_	396.50
Crystal system, space group	Orthorhombic, *P* *n* *a*2_1_
Temperature (K)	100
*a*, *b*, *c* (Å)	8.5797 (7), 14.7450 (13), 16.7720 (14)
*V* (Å^3^)	2121.8 (3)
*Z*	4
Radiation type	Mo *K*α
μ (mm^−1^)	0.08
Crystal size (mm)	0.24 × 0.22 × 0.21

Data collection	
Diffractometer	Bruker SMART CCD 1K area detector
Absorption correction	Multi-scan (*SADABS*; Bruker, 2008 ▸)
*T*_min_, *T*_max_	0.917, 0.984
No. of measured, independent and observed [*I* > 2σ(*I*)] reflections	23380, 6113, 4907
*R*_int_	0.050
(sin θ/λ)_max_ (Å^−1^)	0.703

Refinement	
*R*[*F* ^2^ > 2σ(*F* ^2^)], *wR*(*F* ^2^), *S*	0.056, 0.132, 1.05
No. of reflections	6113
No. of parameters	271
No. of restraints	1
H-atom treatment	H-atom parameters constrained
Δρ_max_, Δρ_min_ (e Å^−3^)	0.33, −0.24

Computer programs: *SMART* (Bruker, 2001 ▸), *SAINT* (Bruker, 2009 ▸), *SHELXS97* (Sheldrick, 2008 ▸), *SHELXL2014* (Sheldrick, 2015 ▸), *PLATON* (Spek, 2009 ▸), *publCIF* (Westrip, 2010 ▸).

## supplementary crystallographic information

### Crystal data

**Table:**

C_28_H_28_O_2_	*D*_x_ = 1.241 Mg m^−^^3^
*M**_r_* = 396.50	Melting point = 413–414 K
Orthorhombic, *P**n**a*2_1_	Mo *K*α radiation, λ = 0.71073 Å
*a* = 8.5797 (7) Å	Cell parameters from 3830 reflections
*b* = 14.7450 (13) Å	θ = 2.4–24.5°
*c* = 16.7720 (14) Å	µ = 0.08 mm^−^^1^
*V* = 2121.8 (3) Å^3^	*T* = 100 K
*Z* = 4	Prism, colourless
*F*(000) = 848	0.24 × 0.22 × 0.21 mm

### Data collection

**Table:**

Bruker SMART CCD 1K area detector diffractometer	4907 reflections with *I* > 2σ(*I*)
Radiation source: sealed X-ray tube	*R*_int_ = 0.050
ω scans	θ_max_ = 30.0°, θ_min_ = 1.8°
Absorption correction: multi-scan (*SADABS*; Bruker, 2008)	*h* = −11→12
*T*_min_ = 0.917, *T*_max_ = 0.984	*k* = −20→20
23380 measured reflections	*l* = −23→23
6113 independent reflections	

### Refinement

**Table:**

Refinement on *F*^2^	Primary atom site location: structure-invariant direct methods
Least-squares matrix: full	Secondary atom site location: difference Fourier map
*R*[*F*^2^ > 2σ(*F*^2^)] = 0.056	Hydrogen site location: inferred from neighbouring sites
*wR*(*F*^2^) = 0.132	H-atom parameters constrained
*S* = 1.05	*w* = 1/[σ^2^(*F*_o_^2^) + (0.0522*P*)^2^ + 1.0029*P*] where *P* = (*F*_o_^2^ + 2*F*_c_^2^)/3
6113 reflections	(Δ/σ)_max_ < 0.001
271 parameters	Δρ_max_ = 0.33 e Å^−^^3^
1 restraint	Δρ_min_ = −0.24 e Å^−^^3^

### Special details

**Table:**

Geometry. All s.u.'s (except the s.u.in the dihedral angle between two l.s. planes) are estimated using the full covariance matrix. The cell s.u.'s are taken into account individually in the estimation of s.u.'s in distances, angles and torsion angles; correlations between s.u.'s in cell parameters are only used when they are defined by crystal symmetry. An approximate (isotropic) treatment of cell s.u.'s is used for estimating s.u.'s involving l.s. planes.

### Fractional atomic coordinates and isotropic or equivalent isotropic displacement parameters (Å^2^)

**Table:**

	*x*	*y*	*z*	*U*_iso_*/*U*_eq_	
O2	0.1174 (2)	0.52918 (13)	0.16577 (13)	0.0218 (4)	
O1	0.1183 (3)	0.75243 (15)	0.14425 (13)	0.0280 (5)	
C18	−0.1447 (3)	0.48947 (19)	0.19621 (18)	0.0196 (5)	
C6	−0.1400 (3)	0.65682 (19)	−0.06165 (19)	0.0225 (6)	
C15	0.1452 (3)	0.6535 (2)	0.29827 (19)	0.0244 (6)	
H15A	0.1763	0.6416	0.3541	0.029*	
H15B	0.1211	0.7189	0.2935	0.029*	
C21	−0.1350 (4)	0.6132 (2)	0.33496 (19)	0.0255 (6)	
H21A	−0.1434	0.6782	0.3492	0.031*	
H21B	−0.1168	0.5787	0.3847	0.031*	
C22	−0.1532 (3)	0.44888 (19)	0.12445 (18)	0.0214 (6)	
H22	−0.0658	0.4572	0.0904	0.026*	
C24	−0.3097 (4)	0.3970 (2)	0.01014 (18)	0.0232 (6)	
H24	−0.2483	0.4358	−0.0224	0.028*	
C12	0.1424 (3)	0.67807 (19)	0.11548 (18)	0.0202 (5)	
C11	0.3598 (3)	0.5667 (2)	0.1080 (2)	0.0245 (6)	
H11A	0.4135	0.5166	0.1360	0.029*	
H11B	0.4368	0.6155	0.0985	0.029*	
C28	−0.3708 (3)	0.3333 (2)	0.1377 (2)	0.0252 (6)	
H28	−0.3509	0.3274	0.1932	0.030*	
C9	0.2076 (4)	0.6050 (2)	−0.01768 (19)	0.0245 (6)	
H9A	0.2822	0.6499	−0.0395	0.029*	
H9B	0.1535	0.5762	−0.0632	0.029*	
C2	−0.3786 (4)	0.6144 (2)	−0.1291 (2)	0.0300 (7)	
H2	−0.4850	0.5966	−0.1267	0.036*	
C19	−0.2720 (3)	0.4892 (2)	0.25860 (18)	0.0231 (6)	
H19A	−0.2474	0.4435	0.3000	0.028*	
H19B	−0.3722	0.4721	0.2335	0.028*	
C10	0.2982 (4)	0.5323 (2)	0.02809 (19)	0.0252 (6)	
H10A	0.2292	0.4796	0.0375	0.030*	
H10B	0.3870	0.5116	−0.0049	0.030*	
C23	−0.2807 (3)	0.39322 (19)	0.09204 (18)	0.0219 (6)	
C13	0.2283 (3)	0.60278 (19)	0.16023 (18)	0.0212 (6)	
C8	0.0891 (4)	0.65388 (19)	0.03317 (18)	0.0218 (6)	
C17	−0.0070 (3)	0.54326 (19)	0.21749 (17)	0.0204 (6)	
C1	−0.2972 (4)	0.6319 (2)	−0.05929 (19)	0.0271 (6)	
H1	−0.3487	0.6269	−0.0094	0.033*	
C14	0.2827 (4)	0.6308 (2)	0.24302 (19)	0.0257 (6)	
H14A	0.3514	0.6844	0.2385	0.031*	
H14B	0.3442	0.5808	0.2668	0.031*	
C25	−0.4265 (4)	0.3451 (2)	−0.0247 (2)	0.0279 (7)	
H25	−0.4440	0.3486	−0.0806	0.033*	
C5	−0.0683 (4)	0.6665 (2)	−0.1359 (2)	0.0264 (6)	
H5	0.0374	0.6853	−0.1387	0.032*	
C4	−0.1497 (4)	0.6491 (2)	−0.2057 (2)	0.0286 (7)	
H4	−0.0992	0.6553	−0.2558	0.034*	
C3	−0.3049 (4)	0.6227 (2)	−0.2025 (2)	0.0307 (7)	
H3	−0.3604	0.6104	−0.2503	0.037*	
C7	−0.0583 (4)	0.67387 (19)	0.01428 (18)	0.0232 (6)	
H7	−0.1179	0.7026	0.0548	0.028*	
C27	−0.4889 (4)	0.2823 (2)	0.1028 (2)	0.0316 (7)	
H27	−0.5506	0.2431	0.1348	0.038*	
C16	0.0016 (3)	0.6001 (2)	0.27985 (19)	0.0230 (6)	
C20	−0.2876 (4)	0.5820 (2)	0.2972 (2)	0.0283 (7)	
H20A	−0.3696	0.5796	0.3387	0.034*	
H20B	−0.3203	0.6267	0.2564	0.034*	
C26	−0.5171 (4)	0.2885 (2)	0.0216 (2)	0.0336 (7)	
H26	−0.5984	0.2539	−0.0020	0.040*	

### Atomic displacement parameters (Å^2^)

**Table:**

	*U*^11^	*U*^22^	*U*^33^	*U*^12^	*U*^13^	*U*^23^
O2	0.0196 (9)	0.0200 (10)	0.0259 (10)	−0.0023 (8)	0.0012 (8)	0.0002 (8)
O1	0.0335 (12)	0.0230 (10)	0.0274 (12)	0.0018 (9)	−0.0003 (9)	−0.0023 (9)
C18	0.0196 (12)	0.0177 (12)	0.0215 (13)	0.0005 (10)	−0.0010 (11)	0.0022 (10)
C6	0.0246 (14)	0.0194 (13)	0.0236 (14)	0.0048 (11)	−0.0015 (12)	−0.0011 (11)
C15	0.0267 (15)	0.0250 (14)	0.0214 (14)	−0.0048 (11)	−0.0046 (12)	−0.0012 (11)
C21	0.0286 (16)	0.0253 (14)	0.0227 (14)	−0.0022 (12)	0.0023 (12)	−0.0006 (12)
C22	0.0202 (12)	0.0200 (13)	0.0239 (15)	−0.0012 (11)	0.0012 (11)	−0.0004 (11)
C24	0.0225 (14)	0.0208 (14)	0.0263 (15)	0.0005 (11)	0.0009 (12)	−0.0011 (11)
C12	0.0188 (12)	0.0195 (13)	0.0222 (14)	−0.0012 (10)	0.0022 (11)	−0.0008 (11)
C11	0.0185 (13)	0.0235 (14)	0.0315 (16)	0.0011 (11)	0.0010 (12)	0.0008 (12)
C28	0.0253 (14)	0.0239 (14)	0.0264 (15)	−0.0033 (11)	−0.0012 (12)	−0.0001 (12)
C9	0.0238 (14)	0.0231 (14)	0.0265 (15)	0.0022 (11)	0.0050 (12)	−0.0028 (12)
C2	0.0276 (15)	0.0295 (16)	0.0328 (17)	0.0010 (12)	−0.0025 (14)	−0.0012 (13)
C19	0.0204 (13)	0.0266 (14)	0.0223 (15)	−0.0027 (11)	0.0006 (11)	0.0001 (12)
C10	0.0212 (13)	0.0245 (14)	0.0300 (16)	0.0024 (11)	0.0051 (12)	−0.0009 (12)
C23	0.0198 (13)	0.0197 (13)	0.0263 (15)	0.0012 (11)	−0.0009 (12)	−0.0025 (11)
C13	0.0172 (12)	0.0205 (13)	0.0258 (14)	−0.0012 (10)	−0.0009 (11)	0.0015 (11)
C8	0.0247 (14)	0.0172 (12)	0.0237 (15)	−0.0010 (10)	0.0035 (12)	−0.0007 (11)
C17	0.0211 (13)	0.0189 (13)	0.0212 (14)	0.0007 (11)	0.0002 (10)	0.0013 (10)
C1	0.0272 (15)	0.0285 (15)	0.0257 (16)	0.0037 (12)	0.0031 (13)	−0.0001 (12)
C14	0.0239 (14)	0.0270 (15)	0.0261 (16)	−0.0051 (12)	−0.0067 (13)	0.0010 (12)
C25	0.0261 (15)	0.0297 (16)	0.0279 (16)	0.0045 (13)	−0.0053 (13)	−0.0045 (13)
C5	0.0290 (16)	0.0228 (14)	0.0275 (15)	0.0017 (11)	0.0023 (13)	0.0002 (12)
C4	0.0361 (17)	0.0271 (15)	0.0228 (15)	0.0056 (13)	0.0028 (13)	0.0018 (12)
C3	0.0377 (18)	0.0276 (16)	0.0267 (16)	0.0051 (13)	−0.0065 (14)	−0.0038 (13)
C7	0.0262 (14)	0.0201 (13)	0.0232 (14)	0.0028 (11)	0.0040 (12)	−0.0018 (11)
C27	0.0294 (15)	0.0269 (15)	0.0386 (19)	−0.0097 (13)	−0.0001 (14)	0.0017 (14)
C16	0.0230 (13)	0.0236 (14)	0.0224 (14)	−0.0011 (11)	−0.0007 (11)	0.0018 (11)
C20	0.0243 (15)	0.0327 (16)	0.0280 (16)	0.0013 (13)	0.0021 (13)	−0.0039 (13)
C26	0.0295 (16)	0.0311 (16)	0.0401 (18)	−0.0068 (13)	−0.0074 (15)	−0.0060 (14)

### Geometric parameters (Å, º)

**Table:**

O2—C17	1.391 (3)	C9—C8	1.510 (4)
O2—C13	1.446 (3)	C9—C10	1.531 (4)
O1—C12	1.216 (4)	C9—H9A	0.9900
C18—C22	1.346 (4)	C9—H9B	0.9900
C18—C17	1.467 (4)	C2—C1	1.387 (5)
C18—C19	1.512 (4)	C2—C3	1.390 (5)
C6—C5	1.396 (4)	C2—H2	0.9500
C6—C1	1.398 (4)	C19—C20	1.520 (4)
C6—C7	1.475 (4)	C19—H19A	0.9900
C15—C16	1.494 (4)	C19—H19B	0.9900
C15—C14	1.537 (4)	C10—H10A	0.9900
C15—H15A	0.9900	C10—H10B	0.9900
C15—H15B	0.9900	C13—C14	1.522 (4)
C21—C16	1.505 (4)	C8—C7	1.337 (4)
C21—C20	1.526 (4)	C17—C16	1.342 (4)
C21—H21A	0.9900	C1—H1	0.9500
C21—H21B	0.9900	C14—H14A	0.9900
C22—C23	1.471 (4)	C14—H14B	0.9900
C22—H22	0.9500	C25—C26	1.380 (5)
C24—C25	1.390 (4)	C25—H25	0.9500
C24—C23	1.397 (4)	C5—C4	1.387 (5)
C24—H24	0.9500	C5—H5	0.9500
C12—C8	1.497 (4)	C4—C3	1.388 (5)
C12—C13	1.529 (4)	C4—H4	0.9500
C11—C13	1.525 (4)	C3—H3	0.9500
C11—C10	1.526 (4)	C7—H7	0.9500
C11—H11A	0.9900	C27—C26	1.386 (5)
C11—H11B	0.9900	C27—H27	0.9500
C28—C27	1.391 (4)	C20—H20A	0.9900
C28—C23	1.402 (4)	C20—H20B	0.9900
C28—H28	0.9500	C26—H26	0.9500
			
C17—O2—C13	115.6 (2)	C9—C10—H10B	109.1
C22—C18—C17	120.1 (3)	H10A—C10—H10B	107.8
C22—C18—C19	125.3 (3)	C24—C23—C28	117.6 (3)
C17—C18—C19	114.5 (3)	C24—C23—C22	118.3 (3)
C5—C6—C1	118.5 (3)	C28—C23—C22	124.0 (3)
C5—C6—C7	122.9 (3)	O2—C13—C14	110.3 (2)
C1—C6—C7	118.6 (3)	O2—C13—C11	105.2 (2)
C16—C15—C14	113.2 (3)	C14—C13—C11	113.1 (2)
C16—C15—H15A	108.9	O2—C13—C12	105.0 (2)
C14—C15—H15A	108.9	C14—C13—C12	113.5 (2)
C16—C15—H15B	108.9	C11—C13—C12	109.1 (2)
C14—C15—H15B	108.9	C7—C8—C12	117.1 (3)
H15A—C15—H15B	107.8	C7—C8—C9	127.5 (3)
C16—C21—C20	112.0 (3)	C12—C8—C9	115.4 (3)
C16—C21—H21A	109.2	C16—C17—O2	122.5 (3)
C20—C21—H21A	109.2	C16—C17—C18	124.8 (3)
C16—C21—H21B	109.2	O2—C17—C18	112.7 (2)
C20—C21—H21B	109.2	C2—C1—C6	120.7 (3)
H21A—C21—H21B	107.9	C2—C1—H1	119.6
C18—C22—C23	128.2 (3)	C6—C1—H1	119.6
C18—C22—H22	115.9	C13—C14—C15	112.0 (2)
C23—C22—H22	115.9	C13—C14—H14A	109.2
C25—C24—C23	121.3 (3)	C15—C14—H14A	109.2
C25—C24—H24	119.3	C13—C14—H14B	109.2
C23—C24—H24	119.3	C15—C14—H14B	109.2
O1—C12—C8	121.9 (3)	H14A—C14—H14B	107.9
O1—C12—C13	122.8 (3)	C26—C25—C24	120.2 (3)
C8—C12—C13	115.3 (2)	C26—C25—H25	119.9
C13—C11—C10	111.3 (2)	C24—C25—H25	119.9
C13—C11—H11A	109.4	C4—C5—C6	120.8 (3)
C10—C11—H11A	109.4	C4—C5—H5	119.6
C13—C11—H11B	109.4	C6—C5—H5	119.6
C10—C11—H11B	109.4	C5—C4—C3	120.2 (3)
H11A—C11—H11B	108.0	C5—C4—H4	119.9
C27—C28—C23	120.8 (3)	C3—C4—H4	119.9
C27—C28—H28	119.6	C2—C3—C4	119.7 (3)
C23—C28—H28	119.6	C2—C3—H3	120.1
C8—C9—C10	113.1 (3)	C4—C3—H3	120.1
C8—C9—H9A	109.0	C8—C7—C6	128.1 (3)
C10—C9—H9A	109.0	C8—C7—H7	116.0
C8—C9—H9B	109.0	C6—C7—H7	116.0
C10—C9—H9B	109.0	C26—C27—C28	120.4 (3)
H9A—C9—H9B	107.8	C26—C27—H27	119.8
C1—C2—C3	120.1 (3)	C28—C27—H27	119.8
C1—C2—H2	120.0	C17—C16—C15	122.4 (3)
C3—C2—H2	120.0	C17—C16—C21	121.1 (3)
C18—C19—C20	110.8 (3)	C15—C16—C21	116.6 (3)
C18—C19—H19A	109.5	C19—C20—C21	111.9 (3)
C20—C19—H19A	109.5	C19—C20—H20A	109.2
C18—C19—H19B	109.5	C21—C20—H20A	109.2
C20—C19—H19B	109.5	C19—C20—H20B	109.2
H19A—C19—H19B	108.1	C21—C20—H20B	109.2
C11—C10—C9	112.6 (2)	H20A—C20—H20B	107.9
C11—C10—H10A	109.1	C25—C26—C27	119.6 (3)
C9—C10—H10A	109.1	C25—C26—H26	120.2
C11—C10—H10B	109.1	C27—C26—H26	120.2
			
C17—C18—C22—C23	−179.5 (3)	C19—C18—C17—C16	−10.4 (4)
C19—C18—C22—C23	−2.9 (5)	C22—C18—C17—O2	−13.6 (4)
C22—C18—C19—C20	−138.5 (3)	C19—C18—C17—O2	169.4 (2)
C17—C18—C19—C20	38.4 (3)	C3—C2—C1—C6	−0.9 (5)
C13—C11—C10—C9	56.7 (3)	C5—C6—C1—C2	2.1 (5)
C8—C9—C10—C11	−47.7 (3)	C7—C6—C1—C2	−179.4 (3)
C25—C24—C23—C28	1.7 (4)	O2—C13—C14—C15	−54.1 (3)
C25—C24—C23—C22	178.9 (3)	C11—C13—C14—C15	−171.6 (2)
C27—C28—C23—C24	−2.5 (4)	C12—C13—C14—C15	63.4 (3)
C27—C28—C23—C22	−179.6 (3)	C16—C15—C14—C13	31.0 (4)
C18—C22—C23—C24	146.7 (3)	C23—C24—C25—C26	0.2 (5)
C18—C22—C23—C28	−36.3 (5)	C1—C6—C5—C4	−2.0 (4)
C17—O2—C13—C14	51.7 (3)	C7—C6—C5—C4	179.6 (3)
C17—O2—C13—C11	173.9 (2)	C6—C5—C4—C3	0.8 (5)
C17—O2—C13—C12	−70.9 (3)	C1—C2—C3—C4	−0.3 (5)
C10—C11—C13—O2	55.0 (3)	C5—C4—C3—C2	0.4 (5)
C10—C11—C13—C14	175.4 (2)	C12—C8—C7—C6	−179.4 (3)
C10—C11—C13—C12	−57.2 (3)	C9—C8—C7—C6	−2.8 (5)
O1—C12—C13—O2	119.4 (3)	C5—C6—C7—C8	−38.5 (5)
C8—C12—C13—O2	−60.5 (3)	C1—C6—C7—C8	143.1 (3)
O1—C12—C13—C14	−1.1 (4)	C23—C28—C27—C26	1.5 (5)
C8—C12—C13—C14	179.0 (3)	O2—C17—C16—C15	0.6 (4)
O1—C12—C13—C11	−128.2 (3)	C18—C17—C16—C15	−179.7 (3)
C8—C12—C13—C11	51.9 (3)	O2—C17—C16—C21	−179.6 (3)
O1—C12—C8—C7	−48.0 (4)	C18—C17—C16—C21	0.2 (5)
C13—C12—C8—C7	131.9 (3)	C14—C15—C16—C17	−4.5 (4)
O1—C12—C8—C9	135.0 (3)	C14—C15—C16—C21	175.6 (3)
C13—C12—C8—C9	−45.1 (3)	C20—C21—C16—C17	−18.7 (4)
C10—C9—C8—C7	−134.9 (3)	C20—C21—C16—C15	161.2 (3)
C10—C9—C8—C12	41.8 (3)	C18—C19—C20—C21	−57.5 (3)
C13—O2—C17—C16	−25.4 (4)	C16—C21—C20—C19	47.1 (4)
C13—O2—C17—C18	154.9 (2)	C24—C25—C26—C27	−1.3 (5)
C22—C18—C17—C16	166.6 (3)	C28—C27—C26—C25	0.4 (5)

### Hydrogen-bond geometry (Å, º)

**Table:**

*D*—H···*A*	*D*—H	H···*A*	*D*···*A*	*D*—H···*A*
C20—H20*B*···O1^i^	0.99	2.64	3.630	175
C11—H11*B*···O1^i^	0.99	2.61	3.521	153

Symmetry code: (i) −*x*, −*y*, *z*+1/2.
